# Association of lung function and blood glucose level: a 10-year study in China

**DOI:** 10.1186/s12890-022-02208-3

**Published:** 2022-11-25

**Authors:** Wei Li, Yi Ning, Yuan Ma, Xinshan Lin, Sailimai Man, Bo Wang, Chen Wang, Ting Yang

**Affiliations:** 1 Department of Pulmonary and Critical Care Medicine, Center of Respiratory Medicine, China-Japan Friendship Hospital, No. 2, East Yinghua Road, Chaoyang District, Beijing, 100029 China; 2 Meinian Institute of Health, Beijing, 100191 China; 3 School of Population Medicine and Public Health, Chinese Academy of Medical Sciences/Peking Union Medical College, Beijing, 100730 China; 4 Peking University Health Science Center Meinian Public Health Institute, Beijing, 100191 China; 5 Department of Epidemiology and Biostatistics, School of Public Health, Peking University, Beijing, 100091 China

**Keywords:** Lung function, Hyperglycemia, Chronic obstructive pulmonary disease, Spirometry, Decline

## Abstract

**Background:**

At present, chronic respiratory diseases are a major burden in terms of morbidity and mortality and are of increasing public health concern in China. Meanwhile, the prevalence of diabetes has increased by more than 10 times over the last 40 years. While a few studies have investigated the association between chronic respiratory diseases and diabetes mellitus, the association is not clear. This study aimed to explore this association and provide evidence.

**Methods:**

In this single-center study, we enrolled participants aged ≥ 20 years undergoing at least two regular health check-ups from 2009 to 2019 at MJ Healthcare Center in Beijing. Each health check-up included physical examination, biochemical tests, a pulmonary function test, a questionnaire. A total of 11,107 adults were included, and cross-sectional and longitudinal analyses were performed.

**Results:**

We found that both prediabetic and diabetic adults had lower lung function than the normal population at baseline, indicating that lung function decline may start from prediabetic status. Quantitatively, with 1-mmol/L increase in fasting plasma glucose level, the forced vital capacity (FVC), forced expiratory volume in 1 s (FEV_1_), FVC% and FEV_1_% lowered by 25 ml, 13 ml, 0.71-1.03%, and 0.46-0.72%, respectively. However, no significant difference was found in the rates for the lung function decline among different baseline diabetes statuses.

**Conclusion:**

People with higher blood glucose level had more severe lung function decline, with decline starting from prediabetic status, but no significant difference was noted in the rate of lung function decline based on different baseline diabetic statuses.

**Supplementary Information:**

The online version contains supplementary material available at 10.1186/s12890-022-02208-3.

## Background

Pulmonary disease is one of the most prevalent diseases in China as well as internationally, contributing as a predominant factor to worldwide disease burden. However, pulmonary function tests have just started to be performed pervasively in China. Recently, several large-scale epidemiological investigations in China revealed that the prevalence of chronic respiratory diseases, such as asthma and chronic obstructive pulmonary disease (COPD), is much higher than expected [1, 2], with 13.7% people aged ≥ 40 years having spirometry-defined COPD [2].

On the other hand, the International Diabetes Federation [3] stated that the prevalence of diabetes in females and males worldwide is 8.4 and 9.1%, respectively. Chronic respiratory disease and diabetes are both pervasive in the general population. Diabetic patients report respiratory symptoms frequently [4], and diabetes mellitus is considered a risk factor for several pulmonary systemic diseases [5, 6], including asthma, COPD, obstructive sleep apnea, pulmonary hypertension, and idiopathic pulmonary fibrosis.

From the end of twentieth century, people have been questioning whether diabetes mellitus influences the pulmonary system and how it affects pulmonary health. Results from several cross-sectional studies suggest that patients with diabetes have lower forced vital capacity (FVC) and forced expiratory volume in 1 s (FEV_1_) than their non-diabetic counterparts [7–11]. However, for the change rate of lung function decline between diabetic and non-diabetic groups, different studies have provided different results [8–12].

To date, evidence on the association between diabetes and pulmonary diseases is conflicting. Therefore, aiming to address this conflicting association with solid proof, we conducted this research using data from 11,107 participants at baseline and prospectively in a longitudinal study. This study aimed to investigate the association between chronic respiratory diseases and diabetes mellitus and to elucidate changes in lung function with transformation of diabetes.

## Methods

### Data source and study population

In our single-center study performed at MJ Healthcare Center in Beijing retrospectively, we enrolled participants aged ≥ 20 years undergoing at least two regular health check-ups from 2009 to 2019. These participants attended one health check-up from 2009 to 2012 and another one or two check-ups from 2013 to 2019. Each health check-up included physical examination, biochemical tests, a pulmonary function test (without bronchodilator) and a questionnaire. Information in the questionnaire included past medical histories, medication use, current health situation, family history, and exercise frequency. Participants were excluded at baseline if they had (1) cardiovascular diseases, (2) chest surgery, (3) previous or current chronic pulmonary diseases (e.g., lung cancer, asthma, pulmonary tuberculosis), and (4) medication history for cardiovascular disease or asthma.

### Definitions of diabetic status

Diagnosis of diabetic status was made according to either of the following three conditions: (1) according to the International Statistical Classification of Diseases and Related Health Problems, 11th revision (ICD-11), codes 5A11, subjects with fasting plasma glucose (FPG) levels ≥ 126 mg/dL (7.0 mmol/L) at the first health check-up; (2) participants who mentioned diabetes in their past medical history in the questionnaire; (3) participants who stated that they took medication for diabetes in the questionnaire.

For both prediabetic and normal individuals, there should be no mention of diabetes or use of medication for diabetes in the questionnaire. Diagnosis of prediabetic status was made based on the FPG level at the first health check-up, with FPG level ≥ 110 mg/dL (6.1 mmol/L) but < 126 mg/dL (7.0 mmol/L) [13]. Diagnosis of normal status was also made based on FPG level, with FPG < 110 mg/dL (6.1 mmol/L).

### Definitions of different lung function status

We grouped participants into Normal, Preserved Ratio Impaired Spirometry (PRISm), COPD groups according to their baseline lung function. PRISm: FEV_1_/FVC ≥ 0.7 and FEV_1_% < 80%. COPD: FEV_1_/FVC < 0.7. These definitions were made based on spirometry without bronchodilator.

### Pulmonary function test performance

In this study, FEV_1_ and FVC were measured using a portable spirometer (CHEST HI-101, Japan), which was calibrated daily with a 1-L syringe. Pulmonary function tests were performed by experienced technicians at MJ Healthcare Center. Each participant completed two spirometry attempts without bronchodilator while seated. If the two readings differed significantly, a third measurement was required [14]. Lung function data were reported in absolute values, a percentage of predict value (% pred) [15] and 1-s rate (FEV_1_/FVC). Spirometry was performed based on recommendations from the Epidemiology Standardization Project [16] and the American Thoracic Society [17]. The quality control and reproducibility were coordinated by a group formed by the Meinian Institute of Health and China-Japan Friendship Hospital. We calculated the ratios of observed to predicted FEV_1_ based on Chinese population references [18].

### Collection and classification of other variables

To stratify our population by age, we divided participants into the following six age groups: 20 ~ 30, 30 ~ 40, 40 ~ 50, 50 ~ 60, 60 ~ 70, and > 70 years. For stratification analysis of body mass index (BMI), according to the Chinese criteria, we divided participants into three groups: BMI < 24 kg/m^2^, 24 ≤ BMI < 28 kg/m^2^ (overweight), and BMI ≥ 28 kg/m^2^ (obese). For stratification analysis of physical activity frequency, the participants were divided into three groups based on their self-reported details in the questionnaire: < 1 time/week, 1–6 times/week, and ≥ 7 times/week. For smoking status, we stratified the participants into the never smoking group, former smoker group, and current smoker group according to their self-reported details in the questionnaire. Hypertension was diagnosed based on previous medical history or anti-hypertension medicine history self-reported by the participants in the questionnaire. Diabetes medication history was also obtained from the questionnaire. Total cholesterol, triglycerides, FPG, and high-density lipoprotein (HDL) data were obtained from biochemical tests.

### Statistical analysis

Differences across groups were compared using ANOVA for continuous variables and χ2 test for categorical variables. Multivariable linear regression analysis was performed to measure the association of FEV_1_, FVC, FEV_1_%, FVC%, FEV_1_/FVC with both FPG level at baseline from 2009 to 2012 and at the time when lung function parameters were measured again, adjusting for age, sex, BMI, smoking status, physical activity frequency, total cholesterol, triglycerides, HDL, hypertension status, and medication use. Participants were excluded if any variable was not available at baseline.

For the longitudinal study, to incorporate correlations for all observations arising from the same person, we employed linear mixed model with correlated measurement errors to analyze the association of diabetic status with lung function parameters. An Unstructured covariance matrix was specified and maximum likelihood was used to estimate the unknown covariance parameters. Only fixed effects were investigated. The subgroup analysis based on the baseline lung function (PRISm, COPD, Normal) was also performed for the association of FPG and lung function through linear regression models and mixed models.

In the trajectory analysis, we defined the outcome of interest and the time variable as FPG and the follow-up time at three time points. Bayesian Information Criterion (BIC) values were used to select best models and we finally fitted 3 groups for the trajectory of FPG with the order of each equation that described the changes over time in each group as quadratic, cubic, and cubic. The linear regression model was applied to investigate the association between FPG trajectories and lung function with the change rate of pulmonary parameters as the response variable. The change rate of pulmonary parameter was calculated as: (pulmonary parameter at the last measurement time – pulmonary parameter at baseline)/ follow-up time.

All analyses were two-tailed, performed using SAS 9.4 (SAS Institute Inc., SAS Campus Drive, Cary, North Carolina 27,513, USA), with *P*-value < 0.05 considered to be statistically significant. The linear mixed model was performed using the Proc Mixed procedure with Repeated statement in the SAS system. The trajectory analysis was performed using the SAS Trajectory Procedure (Proc Traj) [19].

## Results

### Demographic and clinical characteristics

A total of 11,107 adult participants were included in the present study, who underwent a health check-up from 2009 to 2012 and at least one additional check-up in the following 7 years. Among these participants, 10,501 underwent the second check-up from 2013 to 2015, and 5,668 from 2016 to 2019.

The metabolic status of the participants changed over time, with an increase in mean FPG as well as the proportion of hypertension over time. The mean BMI and HDL levels also increased over these years (*p* < 0.001).

FVC and FEV_1_ decreased steadily spanning over 10 years (Table 1), while changes in FEV_1_/FVC were not consistent as other parameters, which showed slight increase over time.

**Table 1:** Demographic and biochemical characteristics of participants at 3 measurement timepoints

	2009–2012 (*N* = 11,107)	2013–2015 (*N* = 10,501)	2016–2019 (*N* = 5,668)	p
Age (years), Mean ± SD	41.5 ± 10.9	45.1 ± 11.0	47.9 ± 11.0	< 0.001
Men, N(%)	6,583(59.3)	6,255(59.6)	3,254(57.4)	0.022
Smoking status, N(%)				< 0.001
No	7,258(69.0)	6,329(70.8)	3,788(72.5)	
Former	527(5.01)	489(5.47)	310(5.94)	
Current	2,739(26.0)	2,121(23.7)	1,124(21.5)	
Physical activity(time/week), N(%)				< 0.001
< 1	1,788(21.7)	1,533(19.2)	463(12.1)	
1–6	3,944(47.9)	3,502(43.9)	1,653(43.4)	
≥ 7	2,496(30.3)	2,951(36.9)	1,697(44.5)	
Hypertension, N(%)	1,651(14.9)	1,748(16.7)	1,172(20.8)	< 0.001
Diabetic status, N(%)				< 0.001
Normal	9,310(83.8)	8,630(82.2)	4,557(80.4)	
Prediabetes	1,106(9.96)	1,049(9.99)	597(10.5)	
Diabetes	692(6.23)	823(7.84)	514(9.07)	
Total cholesterol(mmol/L), Mean ± SD	4.79 ± 0.89	4.74 ± 0.88	4.76 ± 0.88	< 0.001
BMI (kg/m^2^),Mean ± SD	24.1 ± 3.55	24.3 ± 3.42	24.4 ± 3.42	< 0.001
Triglycerides (mmol/L), Median (P25-P75)	1.15(0.78–1.72)	1.17(0.82–1.72)	1.22(0.87–1.78)	0.018
FPG(mmol/L), Mean ± SD	5.66 ± 0.98	5.68 ± 1.08	5.74 ± 1.18	< 0.001
HDL(mmol/L), Median(P25-P75)	1.30(1.09–1.57)	1.30(1.09–1.58)	1.34(1.11–1.63)	< 0.001
Diabetes medication History, N(%)	356(3.21)	474(4.51)	296(5.22)	< 0.001
FVC, Mean ± SD	3.50 ± 0.85	3.46 ± 0.83	3.41 ± 0.83	< 0.001
FEV_1_, Mean ± SD	2.96 ± 0.71	2.95 ± 0.71	2.91 ± 0.73	< 0.001
FVC%, Mean ± SD	99.8 ± 15.3	100 ± 15.1	101 ± 16.7	< 0.001
FEV_1_%, Mean ± SD	93.1 ± 14.5	95.3 ± 13.7	98.3 ± 22.9	< 0.001
FEV_1_/FVC, Mean ± SD	85.0 ± 8.46	85.4 ± 8.64	85.8 ± 8.35	< 0.001

Variables were compared among three timepoints, Chi-square test was applied for categorical variables and Variance analysis was applied for continuous variables

Baseline characteristics of lung function for participants who attended follow-up in different periods are presented in Table S1, showing that participants who underwent the first follow-up in 2013–2015 had similar FPG and lung function parameters at baseline as participants who underwent in both follow-ups in 2013–2015 and 2016–2019.

### Cross-sectional analysis of lung function and FPG

Analysis of lung function parameters based on FPG level tested according to each of the three time points indicated that for both baseline and cross-sectionally, FPG level was negatively associated with both FVC and FEV_1_ (Table 2). At baseline, 1-mmol/L increase in FPG level was associated with a 25-ml (95% confidence interval [CI], -11 ml to 39 ml) decrease in FVC as well as 0.71% (95%CI, -0.34% to 1.08%) decrease in FVC%. Similarly, 1-mmol/L increase in FPG level was associated with a 13-ml (95%CI, -2 ml to 25 ml) decrease in FEV_1_ and 0.46% (95%CI, -0.09% to 0.83%) decrease in FEV_1_%. FPG level was also marginally significantly associated with a decrease in FEV_1_/FVC (*p* = 0.051) at baseline. The association of FPG and lung function parameters at 2013–2015 and 2016–2019 was very similar to the results at baseline.

**Table 2:** The association of fasting plasma glucose with lung function parameters by multiple linear regression at 3 measurement timepoints

Lung function	Cross-sectional FPG^a^		Baseline FPG^b^	
	Estimate (95% CI)	p	Estimate (95% CI)	p
2009–2012 (*N* = 7,506)				
FVC	-0.025(-0.039 ~ -0.011)	< 0.001	/	/
FEV_1_	-0.013(-0.025 ~ -0.002)	0.027	/	/
FVC%	-0.707(-1.077 ~ -0.336)	< 0.001	/	/
FEV_1_%	-0.458(-0.831 ~ -0.086)	0.016	/	/
FEV_1_/FVC	0.209(-0.001 ~ 0.420)	0.051	/	/
2013–2015 (*N* = 7,484)				
FVC	-0.038(-0.051 ~ -0.026)	< 0.001	-0.024(-0.038 ~ -0.010)	0.001
FEV_1_	-0.025(-0.036 ~ -0.015)	< 0.001	-0.012(-0.024 ~ -0.001)	0.037
FVC%	-1.028(-1.354 ~ -0.702)	< 0.001	-0.691(-1.059 ~ -0.323)	< 0.001
FEV_1_%	-0.717(-1.023 ~ -0.410)	< 0.001	-0.456(-0.797 ~ -0.115)	0.009
FEV_1_/FVC	0.233(0.040 ~ 0.426)	0.018	0.224(0.009 ~ 0.440)	0.042
2016–2019 (*N* = 3,744)				
FVC	-0.036(-0.054 ~ -0.019	< 0.001	-0.027(-0.047 ~ -0.006)	0.013
FEV_1_	-0.018(-0.032 ~ -0.003)	0.015	-0.013(-0.030 ~ 0.004)	0.145
FVC%	-1.001(-1.469 ~ -0.532)	< 0.001	-0.707(-1.269 ~ -0.146)	0.014
FEV_1_%	-0.581(-1.037 ~ -0.126)	0.012	-0.362(-0.922 ~ 0.198)	0.205
FEV_1_/FVC	0.416(0.170 ~ 0.663)	< 0.001	0.310(0.011 ~ 0.608)	0.042

^a^ Models adjust for age (continuous), sex (categorical), BMI (continuous), smoking status (categorical), physical activity frequency (categorical), total cholesterol (continuous), triglycerides (continuous), HDL (continuous), hypertension status (categorical) at 3 measurement timepoints

^b ^Models adjust for baseline age (continuous), sex (categorical), BMI (continuous), smoking status (categorical), physical activity frequency (categorical), total cholesterol (continuous), triglycerides (continuous), HDL (continuous), hypertension status (categorical)

/ No data

Further, we investigated the associations of lung function parameters at 2013–2015 and 2016–2019 with the baseline FPG level. The direction of each association with baseline FPG level was the same as that with FPG level at the time when lung function parameters was measured cross-sectionally above.

### Longitudinal analysis of change in lung function and diabetic status

To determine the association of diabetic status and lung function, tests of a total of 27,276 person-time collected in these 10 years were included in Model 1 (Table 3), adjusted for age and sex. Results showed that the diabetic group had lower FVC and FEV_1_ than the normal group (*p* < 0.001). Moreover, the prediabetic group had intermediate FVC and FEV_1_, which were lower than those in the normal group but higher than those in the diabetic group, indicating that lung function decline starts from a prediabetic stage or earlier. This result was further affirmed in Model 2 and Model 3 (Table 3) after adjustment.

**Table 3:** The association of baseline diabetes status, follow-up time with lung function parameters through the mixed model

	Model 1		Model 2		Model 3	
	Estimate (95% CI)	p	Estimate (95% CI)	p	Estimate (95% CI)	p
FVC						
Diabetic status						
Normal	Ref		Ref		Ref	
Prediabetes	-0.117(-0.153 ~ -0.081)	< 0.001	-0.122(-0.165 ~ -0.080)	< 0.001	-0.104(-0.147 ~ -0.06)	< 0.001
Diabetes	-0.188(-0.232 ~ -0.143)	< 0.001	-0.186(-0.239 ~ -0.133)	< 0.001	-0.140(-0.216 ~ -0.063)	< 0.001
Time	-0.011(-0.013 ~ -0.010)	< 0.001	-0.012(-0.014 ~ -0.010)	< 0.001	-0.013(-0.015 ~ -0.011)	< 0.001
FEV_1_						
Diabetic status						
Normal	Ref		Ref		Ref	
Prediabetes	-0.069(-0.099 ~ -0.040)	< 0.001	-0.071(-0.106 ~ -0.037)	< 0.001	-0.057(-0.092 ~ -0.021)	0.002
Diabetes	-0.112(-0.149 ~ -0.076)	< 0.001	-0.103(-0.146 ~ -0.061)	< 0.001	-0.061(-0.122 ~ 0.001)	0.055
Time	-0.006(-0.007 ~ -0.004)	< 0.001	-0.006(-0.007 ~ -0.004)	< 0.001	-0.007(-0.009 ~ -0.005)	< 0.001
FVC%						
Diabetic status						
Normal	Ref		Ref		Ref	
Prediabetes	-2.944(-3.873 ~ -2.015)	< 0.001	-3.104(-4.190 ~ -2.018)	< 0.001	-2.554(-3.677 ~ -1.431)	< 0.001
Diabetes	-4.892(-6.046 ~ -3.739)	< 0.001	-4.924(-6.275 ~ -3.574)	< 0.001	-3.809(-5.771 ~ -1.847)	0.001
Time	0.150(0.102 ~ 0.197)	< 0.001	0.120(0.064 ~ 0.176)	< 0.001	0.091(0.035 ~ 0.147)	0.002
FEV_1_%						
Diabetic status						
Normal	Ref		Ref		Ref	
Prediabetes	-1.994(-2.886 ~ -1.101)	< 0.001	-2.043(-3.100 ~ -0.986)	< 0.001	-1.742(-2.828 ~ -0.655)	0.002
Diabetes	-3.122(-4.230 ~ -2.013)	< 0.001	-3.014(-4.327 ~ -1.701)	< 0.001	-2.48(-4.377 ~ -0.584)	0.010
Time	0.617(0.559 ~ 0.675)	< 0.001	0.616(0.547 ~ 0.685)	< 0.001	0.623(0.561 ~ 0.685)	< 0.001
FEV_1_/FVC						
Diabetic status						
Normal	Ref		Ref		Ref	
Prediabetes	0.786(0.273 ~ 1.300)	0.003	0.809(0.204 ~ 1.415)	0.009	0.78(0.156 ~ 1.405)	0.014
Diabetes	1.341(0.703 ~ 1.979)	< 0.001	1.455(0.704 ~ 2.207)	< 0.001	1.513(0.421 ~ 2.605)	0.007
Time	0.088(0.056 ~ 0.119)	< 0.001	0.105(0.066 ~ 0.0143)	< 0.001	0.098(0.059 ~ 0.137)	< 0.001

Model 1 adjusted for age (continuous), sex (categorical); Model 2 further adjusted for smoking status (categorical), physical activity frequency (categorical); Model 3 further adjusted for body mass index (continuous), high total cholesterol (categorical), low HDL (categorical), high LDL (categorical), HTN status (categorical), diabetes medication history (categorical). All models contained an interaction term of baseline diabetes status and follow-up time, but none shown statistical significance (*p* > 0.05)

**Fig. 1:**
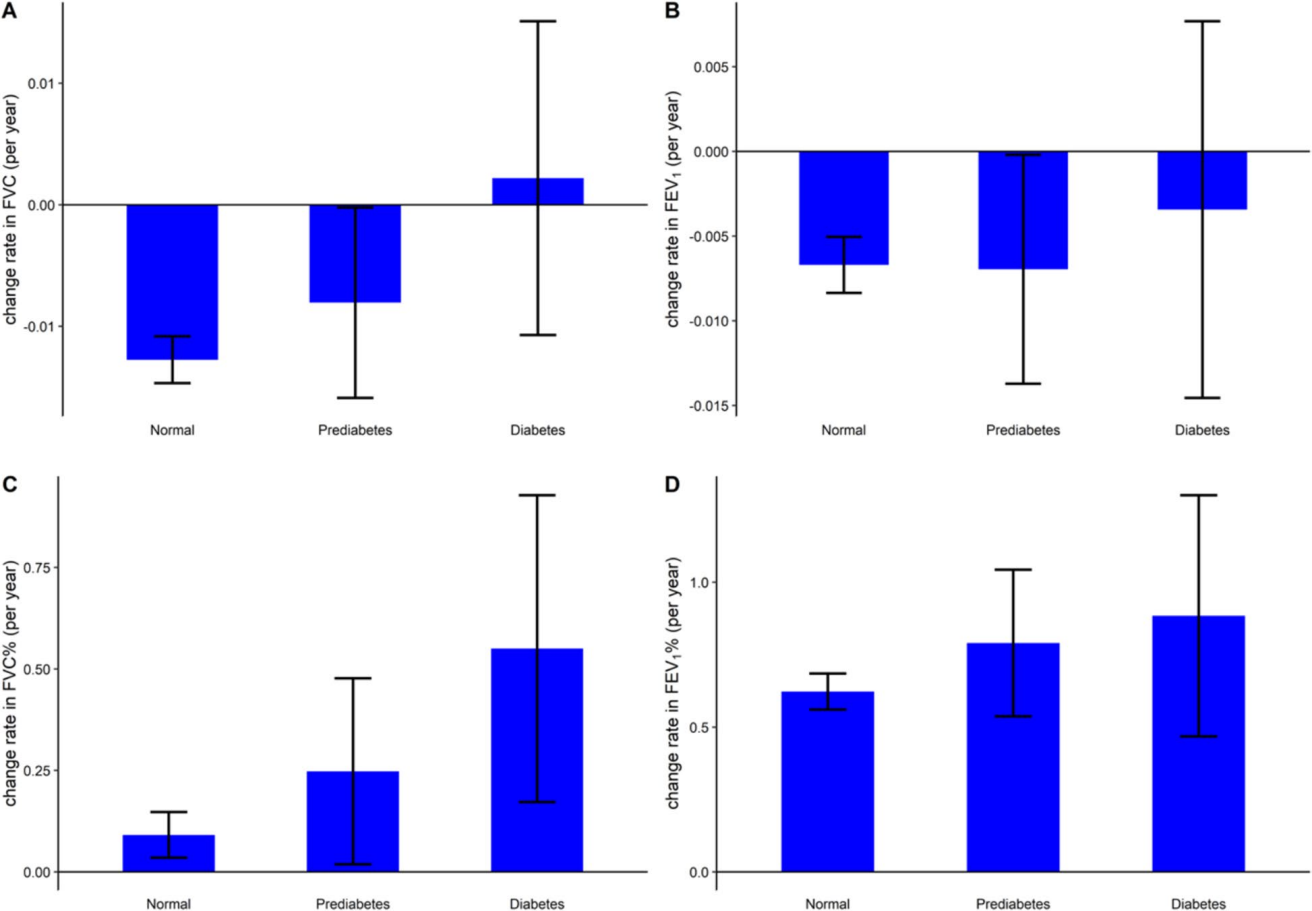
Mean (95% CI) time-dependent change rates in lung function parameters (per year). **a** FVC, **b** FEV_1_, **c** FVC%, **d** FEV_1_%. **a**-**d** were drawn based on the mixed model (full adjusted) with adjustment for baseline covariates, including age (continuous), sex (categorical), smoking status (categorical), physical activity frequency (categorical), body mass index (continuous), high total cholesterol (categorical), low HDL (categorical), high LDL (categorical), HTN status (categorical) and diabetes medication history (categorical)

It is widely acknowledged that time has a negative effect on lung function. Therefore, to study the longitudinal effect of diabetes on lung function, the influence of time should be ruled out. Analysis of the interaction between time and baseline diabetes status (Table 3, Fig. 1) reflected varied rates of FVC, FVC% and FEV_1_% by diabetic status. However, no significance was noted for the interaction, indicating that the rates of lung function decline were similar for different baseline diabetic statuses.

As both blood glucose and lung function can be influenced by factors such as age, smoking status, exercise and other metabolic disorders, stratification analysis was performed for these parameters to identify parameters that participate in the association of baseline diabetic status and lung function. However, the association was not varied by these stratified factors (data not shown).

Trajectory analysis for different blood glucose levels was performed, and the results were similar as those of the cross-sectional analysis and longitudinal analysis above (Figure S1, Table S3 and Table S4).

### Subgroup analysis of change in lung function and diabetic status

To investigate if the baseline lung function status affected this association, we did a subgroup analysis based on different lung function: normal, PRISm and COPD (Table S5, Table S6). Our results showed that there was a trend toward more severe lung function decline (decline of FVC, FEV_1_, FVC%, FEV_1_%) for a worse baseline lung function status. The change rate of lung function decline was more apparent for participants with COPD.

## Discussion

This study examined the association of blood glucose and lung function cross-sectionally and longitudinally.

Previous studies have provided some knowledge on this topic. A few studies suggested that increased incremental decline in FVC and FEV_1_ was associated with increased severity of diabetes [10, 11], but a few longitudinal studies provided opposing results. Results from the Atherosclerosis Risk in Communities (ARIC) Study and the Fremantle Diabetes Study [11, 12] indicated that the speed of pulmonary function decline in diabetic patients was faster than that in non-diabetic controls, while in the Copenhagen City Heart Study and the Normative Aging Study [8, 9], the change rate of lung function decline over time was similar between diabetic and non-diabetic groups. Therefore, the speed of lung function decline in diabetic patients remains inconclusive.

To the best of our knowledge, no study has reported the association between diabetes and pulmonary function in a large-scale Chinese population. The association may be heterogenous by the differences in genetic background.

From our study results, we found that both prediabetic and diabetic adults had lower lung function than the normal population at baseline, indicating that lung function decline may start from a prediabetic status, indicating the inflexion point. Our study not only qualitatively but also quantitively analyzed this association (Table 2). Previously, most of the studies on this topic used baseline FPG level to represent a long-term situation; however, our results doubted the reliability of this method, pointing out that it might underestimate the decline in lung function. We used simultaneous FPG and baseline FPG levels, respectively, to conduct the analysis. Although both methods supported that increased FPG level correlated to more severe lung function decline, the decline was not so definitive using baseline FPG. High blood glucose level may have accumulative function, making it inaccurate to use baseline data to predict the function for a while. Our results suggest that even for predicting long-term lung function change, FPG level examination following each lung function test is necessary for a more exact study.

Our longitudinal analysis tried to answer the intriguing question of whether the decreasing rate of lung function was the same in diabetic patients and normal adults. Our study supported that no significance was found for the decline rate in these two populations based on findings in a large number of participants. This result is surprising as we clearly observed decreased lung function parameters in populations with higher blood glucose at baseline. Three hypotheses help to explain this interesting result. First, the speed of lung function decline may be faster when blood glucose level first becomes abnormal, and with time, it slows down to a “normal” speed, which cannot be differentiated from the decreasing speed in the non-diabetic population. This hypothesis is supported by two studies [8, 20], suggesting that lung function declined faster when adults were first diagnosed with hyperglycemia. Therefore, further investigations are required to determine the inflexion point of lung function decline in diabetic patients. Second, our observation time was not long enough for a mild factor, such as hyperglycemia, to create a difference in lung function decline rate between diabetic and non-diabetic populations. As we studied lung function changes for 3–6 years, future research can prolong the study time to obtained a more affirmative study. Third, as we measured the diabetic status based on baseline FPG level, which only reflected the blood glucose at one time point, it is not a perfect representation of long-term blood glucose level. Abnormal FPG level obtained from health check-ups indicate a need for participants to adjust their lifestyle toward a healthier one. A parameter reflecting the long-term situation of blood glucose is necessary to avoid short-term changes that may change the result.

Although result of our study shows that the magnitude of lung function impairment secondary to diabetes will cause only subclinical abnormalities, a loss of pulmonary reserve capacity may become clinically important in the context of superimposed conditions, such as acute or chronic lung disease. Researchers have started following pulmonary function impairment in a more detailed manner. For example, an epidemiology study of PRISm, which represents slight impairments of both FEV_1_ and FVC, showed that the mortality of it is comparable to that of diabetes [21]. However, for now, PRISm is still not considered as a disease state and the mechanism of it is unclear, and our study may provide clues for it.

This study has some limitations. First, our study was a single-center study in Beijing, and does not represent the entire nation’s population. However, since the pulmonary function test has not been well promoted in China until recently, it is difficult to find both a large number of participants and reliable lung function data. Second, we used FPG level to assess blood glucose, which may lead to a random error. The other parameter, HbA1c, can represent blood glucose long-term; however, it is not an index that is regularly included in biochemical check-ups at most examination centers. Besides, most of the participants who tested for HbA1c had been diagnosed with diabetes, which may lead to a selection bias. Third, we did not analyze the change in lung function in participants whose blood glucose level was high at first and then went back to normal. We are interested in this group because they represent people who care about their health and because this population is becoming larger these days. Analyzing this population helps to provide advice for a better lifestyle. Meanwhile, we did not record the exact time when diabetic patients took their anti-diabetic medication. As a result, we could not analyze the lung function of participants who took the medicine and recovered from hyperglycemia. To perform a basic analysis of medication use, we adjusted diabetes medication history at baseline in Model 3 (Table 3, Fig. 1 and Table S2), and the result was the same as that in the other two models.

## Conclusions

Results from our study suggested that people with higher blood glucose levels had more severe lung function decline, which started from a prediabetic status, suggesting an inflexion point. Moreover, we revealed there was no significance when comparing the rates of lung function decline based on different baseline diabetic statuses.

## Supplementary Information

**Additional file 1:** **Figure S1.** FPG trajectory. **TableS1.** Demographic and biochemicalcharacteristics of participants at baseline. **Table S2.** Demographic and biochemicalcharacteristics of participants by baseline diabetic status at 3 measurementtime points. **Table S3.** Lung function characteristics ofdifferent FPG trajectory groups. **Table S4.** The association of FPG trajectory withlung function change rate through linear regression model. **Table S5.** Subgroup analysis for the association offasting plasma glucose with lung function parameters by multiple linearregression at 3 measurement time points based on the baseline lung function. **Table S6.** Subgroup analysis for the associationof baseline diabetes status, follow-up time with lung function parametersthrough the mixed model according to baseline lung function.

## Data Availability

All data generated and analyzed during this study are included in this published article and its supplementary information files.

## Abbreviations

FPG: Fasting plasma glucose
FVC: Forced vital capacity
FEV_1_: Forced expiratory volume
COPD: Chronic obstructive pulmonary disease
HDL: High-density lipoprotein
PRISm: Preserved ratio impaired spirometry

## References

[1] Huang K, Yang T, Xu J, Yang L, Zhao J, Zhang X, Bai C, Kang J, Ran P, Shen H (2019). Prevalence, risk factors, and management of asthma in China: a national cross-sectional study. Lancet.

[2] Wang C, Xu J, Yang L, Xu Y, Zhang X, Bai C, Kang J, Ran P, Shen H, Wen F (2018). Prevalence and risk factors of chronic obstructive pulmonary disease in China (the China Pulmonary Health [CPH] study): a national cross-sectional study. Lancet.

[3] Cho NH, Shaw JE, Karuranga S, Huang Y, da Rocha Fernandes JD, Ohlrogge AW, Malanda B (2018). IDF Diabetes Atlas: Global estimates of diabetes prevalence for 2017 and projections for 2045. Diabetes Res Clin Pract.

[4] De Santi F, Zoppini G, Locatelli F, Finocchio E, Cappa V, Dauriz M, Verlato G (2017). Type 2 diabetes is associated with an increased prevalence of respiratory symptoms as compared to the general population. BMC Pulm Med.

[5] Ehrlich SF, Quesenberry CP, Van Den Eeden SK, Shan J, Ferrara A (2010). Patients diagnosed with diabetes are at increased risk for asthma, chronic obstructive pulmonary disease, pulmonary fibrosis, and pneumonia but not lung cancer. Diabetes Care.

[6] Khateeb J, Fuchs E, Khamaisi M (2019). Diabetes and lung disease: a neglected relationship. Rev Diabet Stud.

[7] Chance WW, Rhee C, Yilmaz C, Dane DM, Pruneda ML, Raskin P, Hsia CC (2008). Diminished alveolar microvascular reserves in type 2 diabetes reflect systemic microangiopathy. Diabetes Care.

[8] Lange P, Parner J, Schnohr P, Jensen G (2002). Copenhagen City Heart Study: longitudinal analysis of ventilatory capacity in diabetic and nondiabetic adults. Eur Respir J.

[9] Litonjua AA, Lazarus R, Sparrow D, Demolles D, Weiss ST (2005). Lung function in type 2 diabetes: the Normative Aging Study. Respir Med.

[10] Walter RE, Beiser A, Givelber RJ, O'Connor GT, Gottlieb DJ (2003). Association between glycemic state and lung function: the Framingham Heart Study. Am J Respir Crit Care Med.

[11] Yeh HC, Punjabi NM, Wang NY, Pankow JS, Duncan BB, Cox CE, Selvin E, Brancati FL (2008). Cross-sectional and prospective study of lung function in adults with type 2 diabetes: the Atherosclerosis Risk in Communities (ARIC) study. Diabetes Care.

[12] Davis TM, Knuiman M, Kendall P, Vu H, Davis WA (2000). Reduced pulmonary function and its associations in type 2 diabetes: the fremantle diabetes study. Diabetes Res Clin Pract.

[13] World Health Organization, World Health Organization (2006). Definition and diagnosis of diabetes mellitus and intermediate hyperglycemia: report of a WHO/IDF consultation.

[14] Miller MR, Hankinson J, Brusasco V, Burgos F, Casaburi R, Coates A, Crapo R, Enright P, van der Grinten CP, Gustafsson P (2005). Standardisation of spirometry. Eur Respir J.

[15] Lange P, Nyboe J, Jensen G, Schnohr P, Appleyard M (1991). Ventilatory function impairment and risk of cardiovascular death and of fatal or non-fatal myocardial infarction. Eur Respir J.

[16] Ferris BG, Speizer FE, Bishop Y, Prang G, Weener J (1978). Spirometry for an epidemiologic study: deriving optimum summary statistics for each subject. Bull Eur Physiopathol Respir.

[17] Listed N (1979). ATS statement snowbird workshop on standardization of spirometry. Am Rev Respir Dis.

[18] Zheng J, Zhong N (2002). Normative values of pulmonary function testing in Chinese adults. Chin Med J (Engl).

[19] Jones BL, Nagin DS, Roeder K (2001). A SAS procedure based on mixture models for estimating developmental trajectories. Sociol Methods Res.

[20] Yu D, Chen T, Qin R, Cai Y, Jiang Z, Zhao Z, Simmons D (2016). Association between lung capacity and abnormal glucose metabolism: findings from China and Australia. Clin Endocrinol.

[21] Wan ES, Balte P, Schwartz JE, Bhatt SP, Cassano PA, Couper D, Daviglus ML, Dransfield MT, Gharib SA, Jacobs DR, Kalhan R, London SJ, Navas-Acien A, O’Connor GT, Sanders JL, Smith BM, White W, Yende S, Oelsner EC (2021). Association between preserved ratio impaired spirometry and clinical outcomes in US adults. JAMA.

