# Supplementary material for: Association of lung function and blood glucose level: a 10-year study in China
Source: BMC Pulm Med. 2022 Nov 25;22:444. doi: 10.1186/s12890-022-02208-3 (PMC9700934; doi:10.1186/s12890-022-02208-3)
Supplement: Supplementary file 1 — Additional file 1: Figure S1. FPG trajectory. TableS1. Demographic and biochemicalcharacteristics of participants at baseline. Table S2. Demographic and biochemicalcharacteristics of participants by baseline diabetic status at 3 measurementtime points. Table S3. Lung function characteristics ofdifferent FPG trajectory groups. Table S4. The association of FPG trajectory withlung function change rate through linear regression model. Table S5. Subgroup analysis for the association offasting plasma glucose with lung function parameters by multiple linearregression at 3 measurement time points based on the baseline lung function. Table S6. Subgroup analysis for the associationof baseline diabetes status, follow-up time with lung function parametersthrough the mixed model according to baseline lung function. [file 12890_2022_2208_MOESM1_ESM.docx]

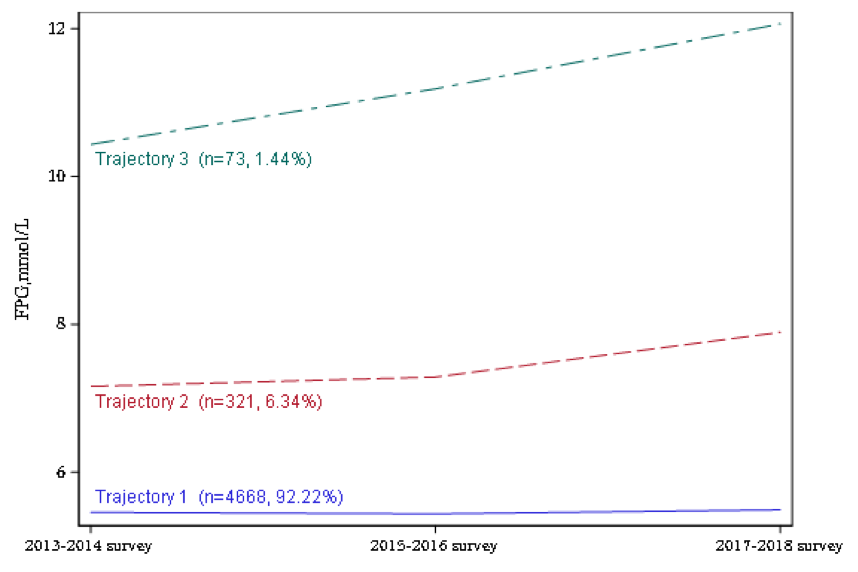


Figure S1. FPG trajectory

TableS1. Demographic and biochemical characteristics of participants at baseline

| Baseline data | Participants in 2009-2012  (N=11,107) | Participants only attended the first follow-up  (N=5,439) | Participants only attended the second follow-up  (N=606) | Participants attended both follow-ups  (N=5,062) | p |
| --- | --- | --- | --- | --- | --- |
| Age(years), Mean±SD | 41.46±10.85 | 41.80±10.91 | 41.96±11.49 | 41.04±10.70 | <0.001 |
| Men, N(%) | 6,583(59.27) | 3,329(61.21) | 328(54.13) | 2,926(57.80) | <0.001 |
| FPG(mmol/L),Mean±SD | 5.66±0.98 | 5.68±1.01 | 5.65±0.83 | 5.64±0.97 | 0.119 |
| FVC,Mean±SD | 3.50±0.85 | 3.52±0.86 | 3.40±0.88 | 3.49±0.83 | 0.003 |
| FEV_1_,Mean±SD | 2.96±0.71 | 2.97±0.71 | 2.90±0.75 | 2.96±0.70 | 0.054 |
| FVCper,Mean±SD | 99.81±15.33 | 99.84±15.44 | 98.75±16.32 | 99.91±15.09 | 0.206 |
| FEV_1_per,Mean±SD | 93.08±14.47 | 93.09±15.49 | 92.51±13.55 | 93.14±13.41 | 0.597 |
| FEV_1_/FVC,Mean±SD | 84.99±8.46 | 84.77±8.41 | 85.46±7.98 | 85.17±8.57 | 0.018 |

Chi-square test was applied for categorical variables and Variance analysis was applied for continuous variables.

Table S2. Demographic and biochemical characteristics of participants by baseline diabetic status at 3 measurement time points

|  | 2009-2012 (N=11,107) | | |  | 2013-2015 (N=10,501) | | |  | 2016-2019 (N=5,668) | | |
| --- | --- | --- | --- | --- | --- | --- | --- | --- | --- | --- | --- |
|  | Normal | Prediabetes | Diabetes |  | Normal | Prediabetes | Diabetes |  | Normal | Prediabetes | Diabetes |
| Age (years), Mean±SD | 39.98±10.41 | 47.93±9.87 | 51.07±9.48 |  | 43.61±10.53 | 51.59±10.07 | 54.53±9.54 |  | 46.47±10.52 | 54.80±10.18 | 57.89±9.56 |
| Men, N(%) | 5,163(55.46) | 869(78.57) | 551(79.62) |  | 4,900(55.70) | 825(78.65) | 530(80.92) |  | 2,583(53.64) | 418(78.28) | 253(79.31) |
| Smoking status, N(%) |  |  |  |  |  |  |  |  |  |  |  |
| No | 6,362(71.98) | 570(54.70) | 325(50.62) |  | 5,523(73.83) | 515(57.41) | 291(51.96) |  | 3,339(75.37) | 277(56.42) | 172(57.14) |
| Former | 378(4.28) | 88(8.45) | 61(9.50) |  | 342(4.57) | 79(8.81) | 68(12.14) |  | 219(4.94) | 54(11.00) | 37(12.29) |
| Current | 2,099(23.75) | 384(36.85) | 256(39.88) |  | 1,616(21.60) | 303(33.78) | 201(35.89) |  | 872(19.68) | 160(32.59) | 92(30.56) |
| Physical activity(time/week), N(%) |  |  |  |  |  |  |  |  |  |  |  |
| <1 | 1,547(22.39) | 161(19.95) | 80(15.66) |  | 1,349(20.15) | 126(15.65) | 58(11.96) |  | 419(12.77) | 25(7.33) | 19(9.90) |
| 1-6 | 3,378(48.89) | 359(44.49) | 207(40.51) |  | 3,023(45.15) | 316(39.25) | 163(33.61) |  | 1,479(45.09) | 112(32.84) | 62(32.29) |
| ≥7 | 1,984(28.72) | 287(35.56) | 224(43.84) |  | 2,323(34.70) | 363(45.09) | 264(54.43) |  | 1,382(42.13) | 204(59.82) | 111(57.81) |
| Hypertension, N(%) | 1,020(10.97) | 341(30.83) | 290(41.91) |  | 1,132(12.89) | 333(31.84) | 283(43.34) |  | 808(16.86) | 207(38.91) | 157(49.22) |
| Diabetes medication History, N(%) | 0(0.00) | 0(0.00) | 356(51.45) |  | 23(0.26) | 43(4.10) | 408(62.29) |  | 25(0.52) | 43(8.05) | 228(71.47) |
| Total cholesterol(mmol/L),  Mean±SD | 4.73±0.86 | 5.11±0.91 | 5.08±1.05 |  | 4.72±0.86 | 4.89±0.89 | 4.81±1.10 |  | 4.76±0.86 | 4.86±0.96 | 4.64±1.01 |
| BMI (kg/m^2^),Mean±SD | 23.66±3.47 | 26.12±3.20 | 26.24±3.19 |  | 23.98±3.39 | 26.01±3.16 | 25.94±3.01 |  | 24.14±3.42 | 26.08±3.18 | 25.70±2.77 |
| Triglycerides (mmol/L),  Median (P25-P75) | 1.07(0.74-1.59) | 1.63(1.18-2.31) | 1.64(1.15-2.51) |  | 1.10(0.78-1.62) | 1.53(1.13-2.18) | 1.53(1.10-2.23) |  | 1.16(0.84-1.71) | 1.58(1.11-2.20) | 1.46(1.04-2.11) |
| FPG(mmol/L),Mean±SD | 5.39±0.34 | 6.33±0.23 | 8.22±2.37 |  | 5.41±0.44 | 6.34±1.01 | 8.31±2.42 |  | 5.45±0.53 | 6.65±1.45 | 8.59±2.47 |
| HDL(mmol/L),Median(P25-P75) | 1.33(1.12-1.60) | 1.18(1.02-1.38) | 1.15(0.99-1.35) |  | 1.32(1.10-1.61) | 1.18(1.00-1.40) | 1.15(0.98-1.39) |  | 1.37(1.13-1.66) | 1.21(1.04-1.43) | 1.21(1.04-1.45) |
| FVC,Mean±SD | 3.53±0.86 | 3.42±0.77 | 3.27±0.73 |  | 3.49±0.84 | 3.40±0.77 | 3.24±0.70 |  | 3.43±0.85 | 3.32±0.78 | 3.17±0.81 |
| FEV_1_,Mean±SD | 2.98±0.72 | 2.89±0.67 | 2.77±0.64 |  | 2.97±0.72 | 2.88±0.68 | 2.74±0.62 |  | 2.94±0.73 | 2.83±0.69 | 2.71±0.70 |
| FVCper,Mean±SD | 100.90±15.31 | 95.29±14.19 | 92.41±14.01 |  | 101.22±14.92 | 96.13±14.53 | 92.99±13.96 |  | 102.11±16.54 | 96.12±15.98 | 93.39±17.19 |
| FEV_1_per,Mean±SD | 93.28±14.57 | 92.45±14.15 | 91.52±13.57 |  | 95.50±13.60 | 94.92±13.93 | 93.56±14.63 |  | 98.37±23.43 | 98.16±20.80 | 96.97±17.24 |
| FEV_1_/FVC,Mean±SD | 85.02±8.47 | 84.76±8.37 | 84.98±8.52 |  | 85.50±8.59 | 84.97±8.98 | 84.75±8.69 |  | 85.82±8.31 | 85.51±8.74 | 85.74±8.32 |

Table S3. Lung function characteristics of different FPG trajectory groups

|  | FPG trajectory1  (N=4668) | FPG trajectory2  (N=321) | FPG trajectory3  (N=73) |
| --- | --- | --- | --- |
| Age (years), Mean±SD | 40.31±10.48 | 49.34±9.26 | 50.90±10.30 |
| Men, N(%) | 2,601 (55.72) | 263 (81.93) | 62 (84.93) |
| **2009-2012** |  |  |  |
| FVC,Mean±SD | 3.51±0.84 | 3.31±0.68 | 3.23±0.67 |
| FEV_1_,Mean±SD | 2.97±0.71 | 2.83±0.60 | 2.78±0.61 |
| FVCper,Mean±SD | 100.59±15.03 | 92.17±13.27 | 90.38±13.78 |
| FEV_1_per,Mean±SD | 93.32±13.39 | 90.92±13.19 | 91.86±14.85 |
| FEV_1_/FVC,Mean±SD | 85.12±8.60 | 85.68±8.29 | 86.34±7.75 |
| **2013-2015** |  |  |  |
| FVC,Mean±SD | 3.46±0.82 | 3.26±0.65 | 3.11±0.65 |
| FEV_1_,Mean±SD | 2.94±0.70 | 2.77±0.58 | 2.71±0.61 |
| FVCper,Mean±SD | 101.00±14.64 | 92.42±12.50 | 88.81±14.34 |
| FEV_1_per,Mean±SD | 95.66±13.31 | 92.69±12.86 | 92.88±16.53 |
| FEV_1_/FVC,Mean±SD | 85.43±8.79 | 85.38±9.23 | 87.05±8.26 |
| **2016-2019** |  |  |  |
| FVC,Mean±SD | 3.43±0.85 | 3.29±0.77 | 3.04±0.69 |
| FEV_1_,Mean±SD | 2.94±0.73 | 2.80±0.67 | 2.69±0.65 |
| FVCper,Mean±SD | 101.94±16.52 | 94.69±16.80 | 88.04±15.21 |
| FEV_1_per,Mean±SD | 98.80±24.09 | 96.61±16.95 | 95.01±16.04 |
| FEV_1_/FVC,Mean±SD | 85.88±8.29 | 85.43±8.74 | 88.43±7.35 |
| **Change rate (per year)** |  |  |  |
| FVC,Mean±SD | -0.01±0.07 | -0.00±0.08 | -0.03±0.07 |
| FEV_1_,Mean±SD | -0.00±0.06 | -0.01±0.07 | -0.01±0.04 |
| FVCper,Mean±SD | 0.18±2.14 | 0.36±2.29 | -0.47±2.01 |
| FEV_1_per,Mean±SD | 0.81±3.17 | 0.82±2.34 | 0.49±1.46 |
| FEV_1_/FVC,Mean±SD | 0.14±1.47 | -0.04±1.57 | 0.45±1.53 |

Table S4. The association of FPG trajectory with lung function change rate through linear regression model

|  | Estimate (95% CI) | p |
| --- | --- | --- |
| FVC |  |  |
| FPG trajectory |  |  |
| Trajectory1 | Ref |  |
| Trajectory 2 | 0.016(0.004-0.027) | 0.006 |
| Trajectory 3 | -0.011(-0.035-0.013) | 0.366 |
| FEV_1_ |  |  |
| FPG trajectory |  |  |
| Trajectory1 | Ref |  |
| Trajectory 2 | 0.002(-0.007-0.012) | 0.624 |
| Trajectory 3 | -0.009(-0.030-0.013) | 0.420 |
| FVC% |  |  |
| FPG trajectory |  |  |
| Trajectory1 | Ref |  |
| Trajectory 2 | 0.430(0.110-0.750) | 0.009 |
| Trajectory 3 | -0.399(-1.101-0.304) | 0.266 |
| FEV_1_% |  |  |
| FPG trajectory |  |  |
| Trajectory1 | Ref |  |
| Trajectory 2 | 0.046(-0.291-0.384) | 0.788 |
| Trajectory 3 | -0.386(-1.128-0.355) | 0.307 |
| FEV_1_/FVC |  |  |
| FPG trajectory |  |  |
| Trajectory1 | Ref |  |
| Trajectory 2 | -0.270(-0.508--0.033) | 0.026 |
| Trajectory 3 | 0.063(-0.458-0.584) | 0.813 |

Notes: All models adjusted for age (continuous), sex (categorical), smoking status (categorical), physical activity frequency (categorical), body mass index (continuous), total cholesterol (continuous), HDL (continuous), LDL (continuous), HTN status (categorical), diabetes medication history (categorical).

Table S5. Subgroup analysis for the association of fasting plasma glucose with lung function parameters by multiple linear regression at 3 measurement time points based on the baseline lung function

| Lung function | Cross-sectional FPG†,  Estimates (95% CI) | | |  | Baseline FPG#,  Estimates (95% CI) | | |
| --- | --- | --- | --- | --- | --- | --- | --- |
|  | Normal  (N=9152) | PRISm (N=1405) | COPD  (N=550) |  | Normal  (N=9152) | PRISm  (N=1405) | COPD  (N=550) |
| 2009-2012 (N=7,506) |  |  |  |  |  |  |  |
| FVC | -0.02(-0.033~-0.008) * | -0.003(-0.025~0.018) | -0.104(-0.201~-0.006) * |  | / | / | / |
| FEV_1_ | -0.012(-0.022~-0.002) * | -0.002(-0.014~0.009) | -0.029(-0.091~0.033) |  | / | / | / |
| FVC% | -0.462(-0.829~-0.094) * | 0.134(-0.492~0.76) | -2.849(-5.671~-0.026) * |  | / | / | / |
| FEV_1_% | -0.338(-0.704~0.029) | 0.031(-0.334~0.395) | -1.254(-3.219~0.711) |  | / | / | / |
| FEV_1_/FVC | 0.143(-0.047~0.333) | -0.023(-0.497~0.451) | 0.861(0.078~1.644) * |  | / | / | / |
| 2013-2015 (N=7,484) |  |  |  |  |  |  |  |
| FVC | -0.03(-0.043~-0.018) * | -0.036(-0.056~-0.015) * | -0.066(-0.137~0.006) |  | -0.016(-0.029~-0.003) * | -0.043(-0.069~-0.017) * | -0.102(-0.192~-0.013) * |
| FEV_1_ | -0.017(-0.027~-0.007) * | -0.023(-0.04~-0.006) * | -0.054(-0.121~0.014) |  | -0.007(-0.017~0.004) | -0.032(-0.053~-0.01) * | -0.049(-0.13~0.033) |
| FVC% | -0.841(-1.201~-0.482) * | -0.976(-1.603~-0.349) * | -1.155(-3.171~0.861) |  | -0.362(-0.748~0.024) | -1.01(-1.824~-0.196) * | -2.489(-5.059~0.082) |
| FEV_1_% | -0.482(-0.805~-0.158) * | -0.707(-1.272~-0.141) * | -1.499(-3.729~0.731) |  | -0.181(-0.528~0.167) | -0.909(-1.628~-0.19) * | -1.718(-4.379~0.942) |
| FEV_1_/FVC | 0.304(0.092~0.517) * | 0.147(-0.259~0.553) | -0.329(-1.72~1.062) |  | 0.205(-0.023~0.434) | 0.058(-0.482~0.597) | 0.732(-0.888~2.352) * |
| 2016-2019 (N=3,744) |  |  |  |  |  |  |  |
| FVC | -0.03(-0.047~-0.012) * | -0.022(-0.05~0.006) | -0.181(-0.351~-0.012) * |  | -0.015(-0.036~0.005) | -0.039(-0.078~0) | -0.189(-0.383~0.004) |
| FEV_1_ | -0.011(-0.026~0.003) | -0.012(-0.035~0.012) | -0.1(-0.249~0.048) |  | -0.004(-0.02~0.013) | -0.026(-0.06~0.007) | -0.156(-0.323~0.011) |
| FVC% | -0.783(-1.3~-0.266) * | -0.598(-1.493~0.297) | -4.718(-9.514~0.078) |  | -0.363(-0.966~0.241) | -0.821(-2.038~0.395) | -5.637(-11.29~0.017) |
| FEV_1_% | -0.308(-0.801~0.185) | -0.457(-1.284~0.369) | -3.312(-8.257~1.632) |  | -0.007(-0.6~0.587) | -0.886(-2.038~0.265) | -5.949(-11.699~-0.199) * |
| FEV_1_/FVC | 0.463(0.19~0.736) * | 0.241(-0.264~0.746) | 1.059(-2.034~4.152) |  | 0.308(-0.014~0.63) | 0.099(-0.622~0.821) | 0.087(-3.213~3.386) |

^†^Models adjust for age (continuous), sex (categorical), BMI (continuous), smoking status (categorical), physical activity frequency (categorical), total cholesterol (continuous), triglycerides (continuous), HDL (continuous), hypertension status (categorical) at 3 measurement time points. For FVC, FEV_1_, body height is further adjusted.

^#^Models adjust for baseline age (continuous), sex (categorical), BMI (continuous), smoking status (categorical), physical activity frequency (categorical), total cholesterol (continuous), triglycerides (continuous), HDL (continuous), hypertension status (categorical). For FVC, FEV1, body height is further adjusted.

*p<0.05, /: no data.

Table S6. Subgroup analysis for the association of baseline diabetes status, follow-up time with lung function parameters through the mixed model according to baseline lung function

|  | Normal (N=9152) | PRISm (N=1405) | COPD (N=550) |
| --- | --- | --- | --- |
| FVC |  |  |  |
| Diabetic status |  |  |  |
| Normal | Ref | Ref | Ref |
| Prediabetes | 0.78(0.156~1.404) * | -0.053(-0.125~0.019) | -0.366(-0.582~-0.149) * |
| Diabetes | 1.513(0.421~2.605) * | -0.072(-0.202~0.059) | -0.466(-0.869~-0.064) * |
| Time | 0.098(0.059~0.137) * | 0.024(0.018~0.029) * | -0.046(-0.058~-0.035) * |
| FEV_1_ |  |  |  |
| Diabetic status |  |  |  |
| Normal | Ref | Ref | Ref |
| Prediabetes | -0.027(-0.056~0.002) | -0.002(-0.043~0.038) | -0.175(-0.324~-0.026) * |
| Diabetes | -0.064(-0.115~-0.013) * | -0.012(-0.085~0.062) | -0.242(-0.519~0.036) |
| Time | -0.016(-0.018~-0.014) * | 0.029(0.024~0.034) * | 0.066(0.055~0.077) * |
| FVCper |  |  |  |
| Diabetic status |  |  |  |
| Normal | Ref | Ref | Ref |
| Prediabetes | -1.842(-2.959~-0.725) * | -0.994(-3.055~1.067) | -7.891(-14.322~-1.461) * |
| Diabetes | -3.305(-5.244~-1.367) * | -1.595(-5.343~2.152) | -13.69(-25.171~-2.209) * |
| Time | 0.004(-0.054~0.063) | 1.136(0.965~1.307) * | -0.816(-1.151~-0.482) * |
| FEV_1_per |  |  |  |
| Diabetic status |  |  |  |
| Normal | Ref | Ref | Ref |
| Prediabetes | -1.289(-2.348~-0.23) * | -0.14(-1.353~1.074) | -5.856(-10.6~-1.113) * |
| Diabetes | -2.533(-4.367~-0.699) * | -0.127(-2.328~2.074) | -8.971(-17.381~-0.562) * |
| Time | 0.353(0.29~0.417) * | 1.722(1.554~1.889) * | 2.965(2.588~3.341) * |
| FEV_1_/FVC |  |  |  |
| Diabetic status |  |  |  |
| Normal | Ref | Ref | Ref |
| Prediabetes | 0.619(0.043~1.195) * | 0.98(-0.541~2.5) | 0.331(-1.582~2.244) |
| Diabetes | 1.281(0.281~2.282) * | 1.398(-1.386~4.182) | 2.083(-1.313~5.479) |
| Time | -0.064(-0.102~-0.026) * | 0.231(0.111~0.351) * | 2.391(2.131~2.651) * |

Notes: Models were full adjusted, variables included: age (continuous), sex (categorical), baseline diabetes status, an interaction term of baseline diabetes status and follow-up time, smoking status (categorical), physical activity frequency (categorical), height (continuous, only for FVC, FEV_1_), body mass index (continuous), high total cholesterol (categorical), low HDL (categorical), high LDL (categorical), HTN status (categorical), diabetes medication history (categorical), an interaction term of diabetes medication history and follow-up time.

*p<0.05
